# Management of essential tremor deep brain stimulation-induced side effects

**DOI:** 10.3389/fnhum.2024.1353150

**Published:** 2024-02-22

**Authors:** Alfonso Enrique Martinez-Nunez, Filipe P. Sarmento, Vyshak Chandra, Christopher William Hess, Justin David Hilliard, Michael S. Okun, Joshua K. Wong

**Affiliations:** ^1^Norman Fixel Institute for Neurological Diseases, Gainesville, FL, United States; ^2^Department of Neurology, University of Florida, Gainesville, FL, United States; ^3^Department of Neurosurgery, University of Florida, Gainesville, FL, United States

**Keywords:** deep brain stimulation, ataxia, dysarthria, dysphagia, gait impairment

## Abstract

Deep brain stimulation (DBS) is an effective surgical therapy for carefully selected patients with medication refractory essential tremor (ET). The most popular anatomical targets for ET DBS are the ventral intermedius nucleus (VIM) of the thalamus, the caudal zona incerta (cZI) and the posterior subthalamic area (PSA). Despite extensive knowledge in DBS programming for tremor suppression, it is not uncommon to experience stimulation induced side effects related to DBS therapy. Dysarthria, dysphagia, ataxia, and gait impairment are common stimulation induced side effects from modulation of brain tissue that surround the target of interest. In this review, we explore current evidence about the etiology of stimulation induced side effects in ET DBS and provide several evidence-based strategies to troubleshoot, reprogram and retain tremor suppression.

## Introduction

Essential tremor (ET) is among the most prevalent hyperkinetic movement disorders with a pooled prevalence estimate of approximately 1% across all ages. Its prevalence increases with advancing age affecting up to 20% of people over 95 years old (Louis and McCreary, 2021). ET is defined as a chronic, insidiously progressive, isolated tremor syndrome characterized by an action tremor of both upper extremities, lasting for a minimum of 3 years in the absence of any other neurological signs such as parkinsonism, ataxia, or dystonia, and may or may not be accompanied by tremor in the head, voice, or lower limbs (Bhatia et al., 2018).

Pharmacological therapy has long been the mainstay of treatment for ET (Deuschl et al., 2011). First-line medications can provide approximately 55–60% mean reduction in tremor amplitude when used as monotherapy (Deuschl et al., 2011). Combined pharmacotherapy can sometimes yield better clinical outcomes (Wagle Shukla, 2022). Up to 55% of patients, however, manifest medication-refractory tremor (Louis, 2005), and thus surgical intervention may be considered in cases with refractory and disabling symptoms (Wagle Shukla, 2022).

Since its FDA approval in 1997, deep brain stimulation (DBS) has been considered a safe and effective therapy for medication refractory ET when applied to carefully selected patients. The location of the implanted lead is a critical determinant in achieving tremor suppression while limiting the manifestation of stimulation-induced side effects. The ventral intermediate nucleus of the thalamus (VIM) has been referred to as a “relay station” in the tremor network, connecting the cerebellum and motor cortex (Schnitzler et al., 2009), and it is the primary target for ET DBS (Benabid et al., 1987, 1991; Koller et al., 1997). The posterior subthalamic area (PSA) has been consistently reported as a target which may also provide optimal tremor suppression (Blomstedt et al., 2009, 2010; Barbe et al., 2011; Fytagoridis et al., 2016), particularly for tremors that are difficult to control with conventional VIM DBS (Kim et al., 2021). The clinical effect of ET DBS has been attributed to the direct modulation of the dentato-rubro-thalamic tract (DRTT) inclusive of the pre-lemniscal radiation and the caudal zona incerta (cZI) (Holslag et al., 2018). In this review we will use the term “thalamic DBS” to include all three of these anatomical regions.

Multiple side effects occur secondary to unintended stimulation of neighboring fiber tracts, which may modulate local and distal regions and neural networks. The most commonly encountered side effects in clinical practice are dysarthria, stimulation-induced ataxia, gait abnormalities, and loss of tremor benefit (habituation). Dysphagia is less common but has been reported. In this review, we will focus on these potential complications and discuss the current available options to reduce both acute and chronic stimulation induced side effects. In this review we discuss programing strategies that have been reported and trialed in the literature. While there is no consensus, this serves as a repository for evidence-based programing in challenging real-world scenarios.

An important concept to keep in mind is that some of the symptoms that we see as stimulation-induced side effects are often part of ET itself. The 2018 consensus classification of tremor (Bhatia et al., 2018 #2) adds a category of “soft signs” or “ET plus” to account for the dysarthria (Biary and Koller, 1987; Barkmeier-Kraemer and Clark, 2017), ataxia (Bhatia et al., 2018), and gait impairment (Fasano et al., 2010) that can be seen in patients with ET as part of the disease. Dysphagia and dysarthria are also possible complications from botulinum toxin injections to treat vocal tremor (Newland et al., 2022). Therefore, careful preoperative clinical evaluation is important to establish each patient’s baseline symptoms and avoid later embarking on a long-winded odyssey to attempt troubleshooting symptoms that are a part of the underlying pathology.

## Dysarthria

Dysarthria stands out as the most common stimulation-induced side effect of thalamic region DBS (Chiu et al., 2020; Lu et al., 2020), with a prevalence reported in the literature ranging from 9% up to 75% (Pahwa et al., 2006; Flora et al., 2010). Despite tremor improvement, thalamic DBS can lead to reduced vocalization and imprecise oral articulation (Mucke et al., 2018).

Speech production is mediated by a network that is centralized around the left laryngeal and orofacial regions of the primary motor cortex. These areas receive inputs from the surrounding premotor, somatosensory, and parietal cortices (Guenther and Vladusich, 2012; Fuertinger et al., 2015). Dysarthria may occur through the spread of current to the corticospinal/corticobulbar tracts and to the DRTT, reflecting either an aggravation of pre-existing cerebellar deficits and/or the involvement of the upper motor neuron (UMN) fibers of the internal capsule (Mucke et al., 2018). Those UMN fibers overlap with the networks associated with tremor benefit following stimulation (Petry-Schmelzer et al., 2021), rendering it challenging to increase stimulation parameters without negatively affecting speech.

Stimulation-induced dysarthria occurs more frequently in those undergoing bilateral DBS (Picillo et al., 2016; Kim et al., 2021). It is also more commonly associated with stimulation on the electrode contacts more dorsally located, usually above the intercommissural line (Barbe et al., 2014; Kim et al., 2021), and those electrodes located relatively lateral (Becker et al., 2017). Spread of current to the medial aspect of the VIM region and to the centromedian and parafascicular thalamic nuclei region may also account for some speech dysfunction following DBS (Crosson, 2013).

Strategies to address stimulation induced dysarthria include proactive pre-operative patient screening for dysarthria along with conscientious lead placement. Staging DBS procedure one lead at a time allows for revaluation of speech and helps in weighting risk vs. benefit of a second lead on speech function in a shared decision-making process. During surgery, microelectrode recordings for target mapping can be used to refine the lead trajectory by ensuring lead placement away from the leg somatotopic representation of the VIM, corresponding to the lateral part of the nucleus, which lies closer to the corticobulbar tract (Garonzik et al., 2002 #102). Macrostimulation from the electrode can further facilitate optimization of the target location by estimating the relative distance from the internal capsule through the stimulation threshold, assessment of clinical benefit, and determination of the presence of stimulation-induced side effects. Nonetheless, in about one-third of patients’ dysarthria will only appear with chronic stimulation (Bot et al., 2018). Therefore, it is important to note that even well-placed electrodes might elicit stimulation-induced adverse events with chronic stimulation. Equally important is to note that in the operating room setting the number of test parameters is limited and this may not translate to the outpatient setting.

When programming a patient with stimulation-induced dysarthria, the initial strategy is usually to decrease the stimulation amplitude (or current density). Although helpful in many cases, this may result in sub-optimal tremor control and should be balanced in a shared decision-making process with the patient (Kim et al., 2021), as most patients prefer the side effects over sub-optimal tremor control (Baizabal-Carvallo et al., 2014; Barbe et al., 2014). Another strategy is to decrease the amplitude one side at a time, starting with the lead that controls the least bothersome hemi-body.

The ability to provide the patient with different programming settings using a handheld patient interface facilitates adjustment of stimulation parameters to fit the context of the situation (e.g., eating vs. speaking). For example, if they were to engage in a public speaking event, they can choose a stimulation setting where they manifest suboptimal tremor control, however, minimal dysarthria is present. In a circumstance where they may be eating a meal, they can choose a stimulation setting where they have complete tremor suppression, but mild dysarthria.

Changing the stimulation site to more ventrally located contacts can reduce dysarthria, considering it is more common when stimulating through dorsal contacts. A bipolar contact configuration is also an option when trying to avoid spreading of current into adjacent structures and undesirable side effects (Kim et al., 2021). When using this strategy, the contact that provides the best tremor control during the monopolar review is chosen as the cathode and the adjacent contact (either dorsal or ventral) is set as the anode. The amplitude should be decreased (down to 1 mA is our practice) and then increase gradually until the side effect comes back to assess the stimulation threshold in this new configuration. Switching the polarity of the selected electrodes might improve effectiveness and provide better tremor control and fewer side effects (Marks, 2011). Another multi-contact technique is using a double monopolar configuration, which was previously described by Kim et al. (2021), in a study that simultaneously targeted the Vim and PSA regions. The double monopolar configuration is a flexible alternative to bipolar configurations through the utilization of current fractionation in some commercially available devices.

Newer generations of DBS hardware may offer options to avoid stimulation-induced dysarthria while maintaining clinical benefit. This can be attempted by applying directional current steering and current fractionation. Essentially these technologies modify the shape of the volume of tissues activated (VTA) around the activated DBS electrode. By shifting the electric field axially along the DBS lead, one can reduce unwanted current spread to adjacent fiber tracts and can decrease stimulation-induced side effects (Rebelo et al., 2018). Rebelo et al. (2018) previously reported on an experimental study significant gains in therapeutic window (91%) and reductions in therapeutic current strength (31%) with stimulation in the “best direction” compared to “omnidirectional stimulation,” without any loss of tremor suppression. Omnidirectional stimulation would be either a full ring or all three segments of a directional lead activated to simulate a complete ring mode. Blume et al. (2017) conducted a prospective, randomized, double-blind study of ten ET patients and observed that directional DBS provided a larger “therapeutic window,” mainly due to lower therapeutic thresholds but not a higher threshold for side effects; therapeutic window is typically defined as the range of stimulation parameters that provides improvement of tremor without causing stimulation-induced side effects. Directional DBS was equally effective as the standard omnidirectional DBS for tremor suppression, and this was not associated with higher energy consumption (Bruno et al., 2021). Though it may be tempting to assume directional DBS is superior to ring mode or omnidirectional DBS, there are few comparison studies and drawing firm conclusions can be tricky.

Interleaving stimulation (ILS) is another useful technique for troubleshooting stimulation-induced dysarthria. This programming method implements two spatially distinct stimulation configurations on the same DBS lead, and it applies the settings in a temporally alternating sequence (Wagle Shukla et al., 2017). Barbe et al. (2014) tested an individualized ILS setting, shifting current from the most effective contact to the immediately dorsal located contact, making this another option to reduce stimulation-induced dysarthria while maintaining tremor control if other strategies have not been successful. Though effective, ILS can also reduce IPG life span over time.

**FIGURE 1:**
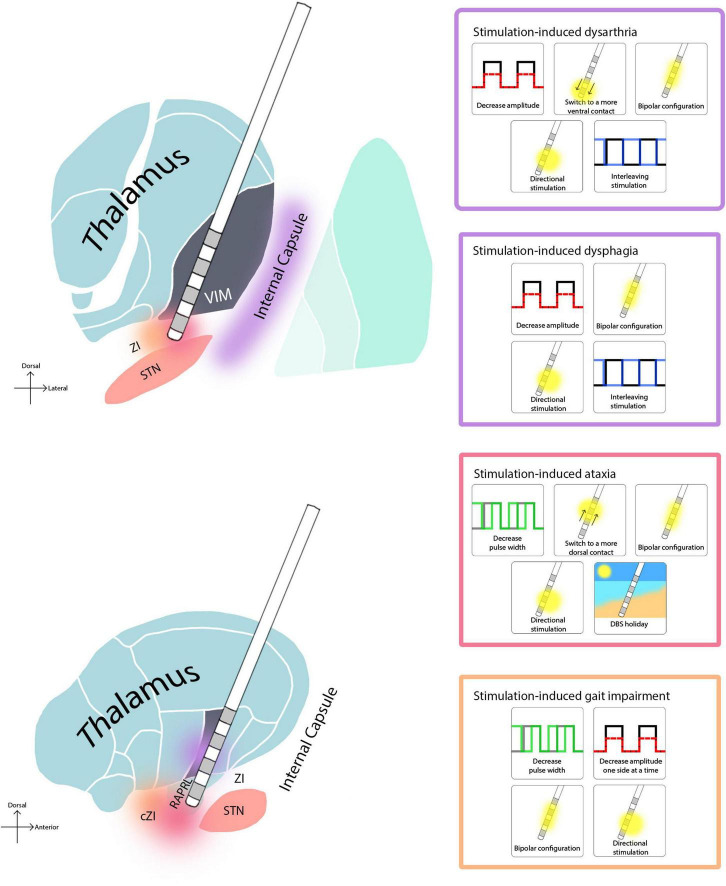
Regions associated with stimulation-induced side effects in thalamic DBS, and troubleshooting options. Since there is no side-to-side comparison of these strategies, we list them in ascending order of complexity.

**FIGURE 2:**
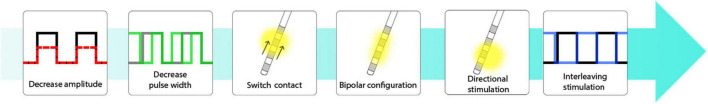
Reprogramming strategies sorted in increasing order of complexity. Since there is no head-to-head comparison of how effective these interventions are, we recommend implementing them in order of simplicity.

Lastly, turning off stimulation at night, or decreasing the amplitude on only one side (Patel et al., 2014; Contarino et al., 2017) may in some select cases reduce dysarthria that might arise as a result of chronic stimulation. A summary of these strategies is depicted in Figure 1. Given that these programing strategies have not been compared in head-to-head studies, there is no evidence to demonstrate that one strategy is more effective than another. Therefore, we recommend implementing them in order of the simplest strategy to the most complex. We present Figure 2 as a protocol in this order for the programmer to have a sequence to follow when cases become complex, and a systematic approach is warranted.

## Dysphagia

The prevalence of stimulation-induced dysphagia is not well documented in the literature. A study that used fiberoptic endoscopy demonstrated some degree of dysphagia in 12/12 thalamic DBS treated ET patients (Lapa et al., 2020). Dysphagia significantly improved in all patients after stimulation was turned off, with a reported mean improvement of 80% in the dysphagia score. The study was a small case series and given that dysphagia is not a common complaint post-thalamic DBS, we would advise caution in overinterpretation. As to the potential mechanism that leads to residual dysphagia, there are many possibilities including the implantation effect, spread of stimulation to corticobulbar fibers, suboptimal lead placement or a combination of a surgical effect plus disease progression. Authors have also postulated a “lingering neural network change” in dysphagia and stimulation-induced ataxia, however, this seems less likely. Finally, it is always important to investigate other potential underlying swallowing pathologies (Lapa et al., 2020).

Both cerebellar and corticobulbar fibers have been posited to play an important role in the process of swallowing. Corticobulbar fibers connect the motor cortex to the cranial nerve nuclei, which innervate the swallowing musculature (Lapa et al., 2020). There is a substantial neuroanatomical overlap of structures involved in the control and execution of speech and swallowing. It is this overlap that has led to speculation that both spread of current into the internal capsule or alternatively interference with the cerebellar network might impact swallowing physiology in a similar manner as in stimulation-induced dysarthria (Hamdy et al., 1996, 1997; Jayasekeran et al., 2011).

The options for troubleshooting stimulation-induced dysphagia are similar to those employed for stimulation-induced dysarthria. Brain imaging is critical to assess the anatomical relation of each contact with the surrounding structures, and “on and off DBS” testing during a barium swallow study can help to define the extent that stimulation is responsible for the acute issue. Reductions in pulse width and/or in amplitude may help swallowing, however, may also worsen tremor control. Bipolar stimulation settings, interleaved stimulation (Barbe et al., 2014), and/or current steering (Barbe et al., 2014; Bruno et al., 2021) may all be tried. Finally, brain imaging, on/off barium swallow testing and programming may in some cases not provide a path for conservative management. In these cases, a revision of the DBS lead should be considered and when retargeting the team should consider trajectory as well as lead location. For a summary of these strategies see Figure 1.

## Ataxia

Stimulation-induced ataxia has been estimated to occur in 35% of patients with thalamic DBS (Chiu et al., 2020) and it has been shown to be acutely “inducible” in almost all patients in the operating room if enough current density is delivered to the thalamic target region (Groppa et al., 2014). Acute ataxia can similarly be reproduced in the clinic setting. Stimulation induced ataxia may impact the limbs, trunk, or features of gait.

Ataxia may also develop insidiously over many years following implantation. The average time has been reported to be approximately 5 years postoperatively, and in small series it is more common in older patients and in patients with a shorter disease duration at the time of DBS implantation (Chiu et al., 2020). The most common types of chronic ataxia presentations are appendicular ataxia (92%) and gait ataxia (44%) (Chiu et al., 2020).

Acute stimulation-induced ataxia most commonly arises following stimulation of the inferior aspect of the VIM and superior aspect of the zona incerta (Hidding et al., 2019). Proposed mechanisms for DBS-induced ataxia have been hypothesized to be related to antidromic stimulation of the cerebellar nodule via the uncinate tract from the subthalamic area (Reich et al., 2016), as well as secondary to plasticity changes in the cerebellum (Fasano and Helmich, 2019), possibly involving the stimulation of fibers to and from the red nucleus and inferior olive and/or fibers originating from interpositus nucleus, bundled within the dentatothalamic fibers (Elble, 2014; Groppa et al., 2014).

Acute stimulation-induced ataxia is now viewed as a circuit disorder by many experts and thus may be due to functional disruption of cerebello-thalamo-cortical networks (Garcia et al., 2003; Fasano et al., 2010; Groppa et al., 2014). Models which have calculated the VTA have shown that ventrocaudal stimulation in the subthalamic area corresponds with more significant gait ataxia and correlates with position emission tomography (PET) changes in which hypermetabolism in the cerebellar nodule increases as stimulation-induced gait ataxia worsens. These effects tend to normalize by approximately 72 h after stimulation is deactivated (Reich et al., 2016). This finding suggests that stimulation-induced ataxia may be reversible with programming or discontinuation of the electrical current.

Groppa et al. (2014) speculates that when we disrupt cerebello-thalamic input with therapeutic stimulation, a second pathway “compensates” for the information lost. Furthermore, he proposes that ataxia occurs when there is modulation of that “secondary pathway” and when the compensatory mechanism is inhibited (Groppa et al., 2014). The tract (if there is a specific tract) responsible for stimulation-induced ataxia has not been clearly identified or agreed upon. Some authors have proposed the ascending limb of the uncinate fasciculus present in the subthalamic area as the critical tract (Reich et al., 2016). This pathway connects efferent fibers from the deep cerebellar nuclei to the thalamus (Elsen et al., 2013; Fine et al., 2014). Finally, a few genetic subtypes of ataxia have been associated with axonal loss in the uncinate fasciculus providing further evidence to support this notion (Stezin et al., 2021).

Fasano and Helmich (2019) recently demonstrated the impact of acute thalamic DBS on gait ataxia in patients with ET, showing improvement with therapeutic stimulation and deterioration following supra-therapeutic stimulation (defined by increasing the amplitude and pulse width until decomposition of movement in the finger to nose test). This finding suggests that cerebellar dysfunction in these patients may be differentially modulated with optimal versus supra-therapeutic stimulation, possibly through recruitment of a different fiber system other than the DRTT based on chronaxie characteristics (Groppa et al., 2014). This observation has in general been translated into small studies using lower pulse widths of 30 μs (Choe et al., 2018) and 40 μs (Moldovan et al., 2018), which have demonstrated a reduction in acute stimulation-induced ataxia while retaining tremor benefit. An important caution is that chronaxie estimates using extracellular stimulation have been difficult to interpret versus regional neuroanatomy and thus must be interpreted with caution (Grill et al., 2005; Elble, 2014).

For troubleshooting, a common core principle is that strategies should include reprogramming trials that last at least a week or two to adequately evaluate delayed benefits and waning of benefits. The evaluation usually begins by repeating a monopolar review. A monopolar review is when the clinician programs for benefit and side effect at each contact and uses this information to guide potential strategies. Potential reprogramming strategies should in general aim to move the stimulation field, in a relative sense, away from cerebellar fibers. Moving active contact(s) dorsally is one such strategy. Another is using a bipolar configuration to narrow the VTA (Contarino et al., 2017). In select cases, current steering may lead to less ataxia compared to standard omni-directional stimulation (Bruno et al., 2021; Hidding et al., 2022; Roque et al., 2022). In newer generation hardware, a lower pulse width (less than 60 μs) (Choe et al., 2018; Moldovan et al., 2018) can also be attempted. Differences in axon diameters and chronaxies can be used by shortening pulse widths to achieve more selective activation of cerebellothalamic fibers, which may mediate tremor control, with less induction of ataxia (Kroneberg et al., 2019). If the monopolar review reveals low thresholds at active contacts and failure to maintain benefit, lead re-implantation may be considered.

**FIGURE 3:**
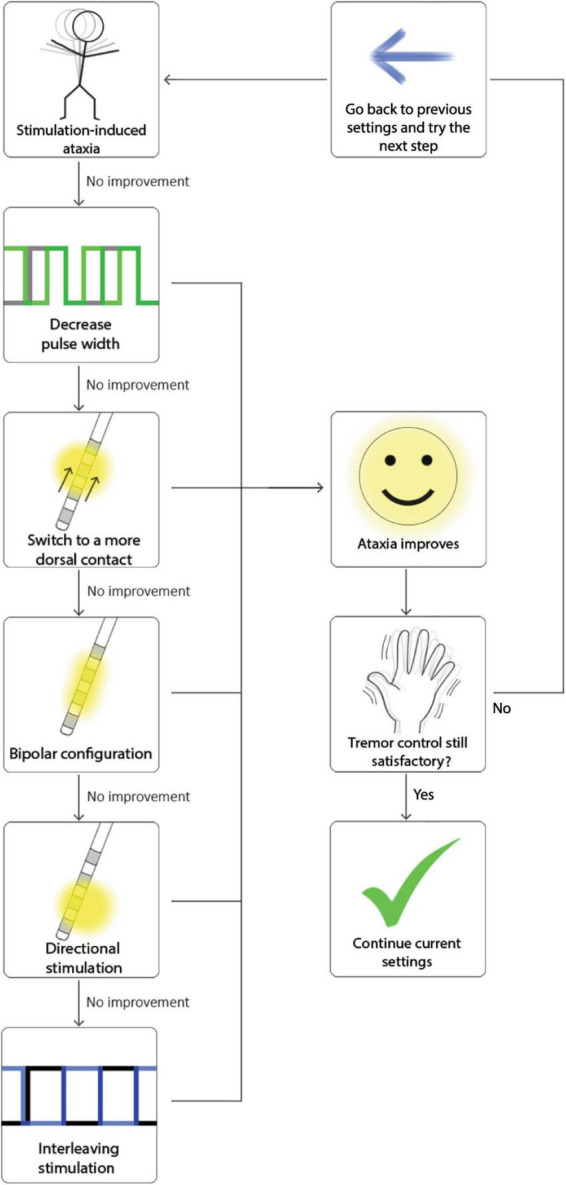
Example of how to troubleshoot ataxia implementing the strategies in Figure 1 and using the order proposed in Figure 2.

In addition to reprogramming, another strategy is turning off the stimulation at night, as this may potentially impact the onset of stimulation induced ataxia (Rebelo et al., 2018). Finally, if ataxia or clumsiness emerges slowly and chronically, this effect is more likely disease progression and is less amenable to programming strategies. For a visual summary of these strategies see Figure 1, and for an example of their implementation see Figure 3.

## Gait and balance impairment

Impairments of balance and gait in patients with ET were reported by clinicians long before a formal association was explored or DBS was developed (Critchley, 1972; Singer et al., 1994). It has been shown in ET that there may be difficulties with tandem gait, balance confidence, and require significantly greater time to perform the Timed Up-and-Go relative to controls (Earhart et al., 2009). Worsening of pre-existing or new-onset gait and balance impairments following thalamic DBS affects between 5–50% of patients with ET (Benabid et al., 1998; Pahwa et al., 2006; Earhart et al., 2009; Kroneberg et al., 2019). However, others have presented contradictory evidence showing that DBS has no adverse effect on gait and balance in unilateral and bilateral stimulation (Earhart et al., 2009; Ramirez-Zamora et al., 2016). Age, disease severity and preoperative gait difficulties are considered risk factors for gait and balance impairment following DBS surgery (Newland et al., 2022). Despite adequate tremor control, patients may experience changes in gait either as an early acute or as a delayed side effect following DBS activation.

The acute phenomenon of gait and balance impairment with DBS is believed to be caused by stimulation-induced network dysfunction, specifically from antidromic cerebellar activation. However, the exact mechanism remains elusive (Earhart et al., 2009). More posterior and medial stimulation are believed to activate cerebellothalamic tracts, leading to gait disturbance, especially when stimulating below the ICL (Murata et al., 2003; Kim et al., 2021). The persistence of stimulation-induced gait impairment after turning off stimulation has led to the suggestion of a possible “microlesioned effect” as the cause (Roemmich et al., 2019). Temporal circuit plasticity is also a possible etiology if considering the 72-h delay observed in some cases for the gait to improve following discontinuation of DBS (Earhart et al., 2009).

Management of imbalance can be challenging, as reduction in stimulation parameters commonly leads to tremor recurrence or has no effect on balance (Ramirez-Zamora et al., 2016). Considering the probable etiological overlay between gait/balance impairments and the cerebellar ataxic features described in the “Stimulation-induced ataxia” section, the common core strategies to troubleshoot this side effect are the same as described above for ataxia.

Besides the previously suggested strategies, dual VIM + PSA stimulation have been reported as an efficacious strategy to mitigate gait disturbances by Kim et al. (2021), and reducing stimulation frequency from 170–185 Hz to 130 Hz after optimizing tremor control was also effective in improving balance difficulties while maintaining tremor control as demonstrated by Ramirez-Zamora et al. (2016).

## Habituation versus disease progression

Habituation to stimulation, also referred to as “tolerance” (Benabid et al., 1996), is a hotly debated topic in the field of neuromodulation. Chronic high intensity stimulation has been hypothesized to induce detrimental plastic effects on tremor networks over time that may ultimately lead to decreased symptomatic control (Pilitsis et al., 2008). Alternatively, sparse post-mortem findings mildly support a biological adaptation to stimulation (Peters and Tisch, 2021). There is much debate about natural disease progression and habituation in the gradual loss of DBS efficacy over time. The characterization and quantification of the amount of overall worsening that is possibly due to loss of the stimulation effect, plastic effects or disease progression is challenging (Peters and Tisch, 2021).

In some studies, “habituation” has been found to occur in as many as 73% of patients with a mean follow-up of 56 months (Shih et al., 2013), and as early as 10 weeks post-implantation (Barbe et al., 2011). Another study showed that non-DBS treated ET controls had similar tremor worsening over time than those tracked with the DBS on and off. Favilla et al. (2012) made a strong argument that much of the chronic worsening in ET DBS may be related to disease progression. A retrospective analysis conducted by Tsuboi et al. (2020) assessed the long-term effects of VIM DBS in 97 patients with essential or dystonic tremor for as long as 13 years in some patients. In this study there were sustained benefits for both types of tremors.

Disease progression, lead misplacement, and suboptimal stimulation are the most commonly cited causes of gradual loss of efficacy in thalamic DBS (Blomstedt et al., 2007; Rodriguez Cruz et al., 2016; Fasano and Helmich, 2019), however, the placebo effect, loss of the microlesional effect (Benabid et al., 1987; Sydow et al., 2003), tissue impedance changes (Benabid et al., 1991; Boockvar et al., 2000; DiLorenzo et al., 2014), and stimulation-induced side effects may also be causes. Initial misdiagnosis has also been encountered in patients with tremor, including patients with multiple sclerosis, fragile X-associated tremor/ataxia syndrome (Artusi et al., 2018), and demyelinating neuropathy (Patel et al., 2014) rather that ET.

Whether the effect observed is called habituation or disease progression, overcoming it is challenging. Worsening typically manifests as a loss of initial DBS benefit in reducing tremor, and simply increasing the stimulation current may worsen tremor severity or induce stimulation-related side effects. Changing the active lead and stimulation parameters may lead to better tremor control, although these effects may not be sustained (Barbe et al., 2011). Studies have compared standard stimulation to weekly or daily rotating stimulation with mixed effects (Barbe et al., 2011; Seier et al., 2018; Petry-Schmelzer et al., 2019).

The initial evaluation should ideally include ruling out the presence of iatrogenic tremor caused by excessive stimulation (Fasano and Helmich, 2019). Fasano suggests reductions in pulse widths, followed by reduction in amplitude. Once stimulation-induced cerebellar tremor is ruled out, the next step should be increasing the frequency and then increasing the amplitude (Fasano and Helmich, 2019). Our experience is that once progression of ataxia and tremor have set in, there are limited management strategies to mitigate them.

Clinicians may attempt in challenging cases to widen the therapeutic window and allow for higher stimulation amplitudes. These strategies include using a bipolar lead configuration, switching the polarity of an existing bipolar setting, applying interleaved stimulation, utilizing directional leads, or shortening pulse widths. In some rare cases, adding an additional stimulation contact may also have a possible benefit, particularly if it is placed near the border of the thalamus (Picillo et al., 2016; Fasano and Helmich, 2019). Another suggested strategy is closed loop DBS (Kronenbuerger et al., 2006; Yamamoto et al., 2013).

There might be a potentially maladaptive response to long-term stimulation that may lead to some of the stimulation induced side-effects (Contarino et al., 2017). Therefore, having some time off the stimulation has been studied in the form of DBS holidays (Garcia Ruiz et al., 2001) or turning the stimulation off at night (Hariz et al., 1999). It should be noted that DBS holidays have been associated in some series with a prominent and debilitating rebound tremor despite symptomatic improvements. Paschen et al. (2019) recently observed that this rebound phenomenon tends to reach a plateau 30–60 min after DBS has been turned off, though the authors note that it is not always present. Furthermore, when utilized, the ideal duration of the DBS holiday is not known and, in our practice as in most expert practices, we do not recommend a DBS holiday.

The last option for a reduction in DBS benefit over time would be surgical revision. This can involve removal and repositioning of a lead or adding a new lead without removing the old one, often referred to as “rescue surgery” (Koller et al., 2001). Secondary leads have been added to many targets including the Vop, PSA or cZI (Yu et al., 2009; Oliveria et al., 2017). A key consideration for repeating surgery is the trajectory (more vertical may be useful for head tremor and helpful to avoid tracts leading to adverse events). Another consideration is whether the issue is ataxia or tremor. This therapy may be best when treating ataxia. Finally, distal tremor is easier to capture than proximal tremor, so a careful examination prior to making any surgical decisions should be pursued.

## Conclusion

Stimulation-induced side effects are common in ET patients treated with thalamic DBS. Additionally most ET DBS patients experience some progression of disease or worsening of their tremor over time. As we learn more about the implicated brain networks in ET, we can potentially build several strategies to increase the therapeutic window for stimulation management without compromising tremor control. Understanding the pathophysiology of these ET DBS side effects will likely empower refined programming strategies and improved surgical planning.

## Author contributions

AM-N: Visualization, Writing—original draft, Writing—review and editing. FS: Writing—original draft, Writing—review and editing. VC: Writing—review and editing. CH: Writing—review and editing. JH: Writing—review and editing. MO: Writing—review and editing. JW: Writing—review and editing.
